# Standard development at the Human Variome Project

**DOI:** 10.1093/database/bav024

**Published:** 2015-03-26

**Authors:** Timothy D. Smith, Mauno Vihinen

**Affiliations:** ^1^Human Variome Project, Level 5, 234 Queensberry Street, The University of Melbourne, Victoria, 3010, Australia and ^2^Department of Experimental Medical Science, BMC D10, Lund University, SE-22184 Lund, Sweden

## Abstract

The Human Variome Project (HVP) is a world organization working towards facilitating the collection, curation, interpretation and free and open sharing of genetic variation information. A key component of HVP activities is the development of standards and guidelines. HVP Standards are systems, procedures and technologies that the HVP Consortium has determined must be used by HVP-affiliated data sharing infrastructure and should be used by the broader community. HVP guidelines are considered to be beneficial for HVP affiliated data sharing infrastructure and the broader community to adopt. The HVP also maintains a process for assessing systems, processes and tools that implement HVP Standards and Guidelines. Recommended System Status is an accreditation process designed to encourage the adoption of HVP Standards and Guidelines. Here, we describe the HVP standards development process and discuss the accepted standards, guidelines and recommended systems as well as those under acceptance. Certain HVP Standards and Guidelines are already widely adopted by the community and there are committed users for the others.

## Background

The Human Variome Project (HVP, http://www.human variomeproject.org/) is an international non-governmental organization that is working towards the integration of the collection, curation, interpretation and sharing of information on human genetic variation into routine clinical practice and research. The ultimate goal is to see all information from all countries on genetic variation and its effect on human health shared freely and openly. The HVP provides a central coordinating function for numerous national and international efforts. The HVP includes a growing consortium of over 1100 individual researchers, healthcare professionals and policy makers and organizations from 81 countries.

Rather than developing and operating data sharing resources itself, the HVP assists its Consortium Members and affiliated initiatives to develop and maintain the necessary systems and infrastructure to support global-scale genomic knowledge sharing by (i) collaboratively developing technical standards and harmonized, common approaches and taking into account the ethical, legal and social requirements of both the data sources and consumers, (ii) coordinating an international platform to facilitate discussion of genomics in global health to foster necessary professional interaction and debate in the area of genomics, global health and service delivery and safety, (iii) linking world leading professionals and institutions in all parts of the world and (iv) establishing a global evidence base for knowledge sharing in medical genetics and genomics and bringing relevant issues to the attention of Ministries of Health, Science and Technology and Education. To achieve this, the HVP Consortium works collaboratively to define international standards and guidelines that describe best-practice methodology in each of these areas, which can then be utilized in the implementation of specific systems and processes.

**Figure 1.**
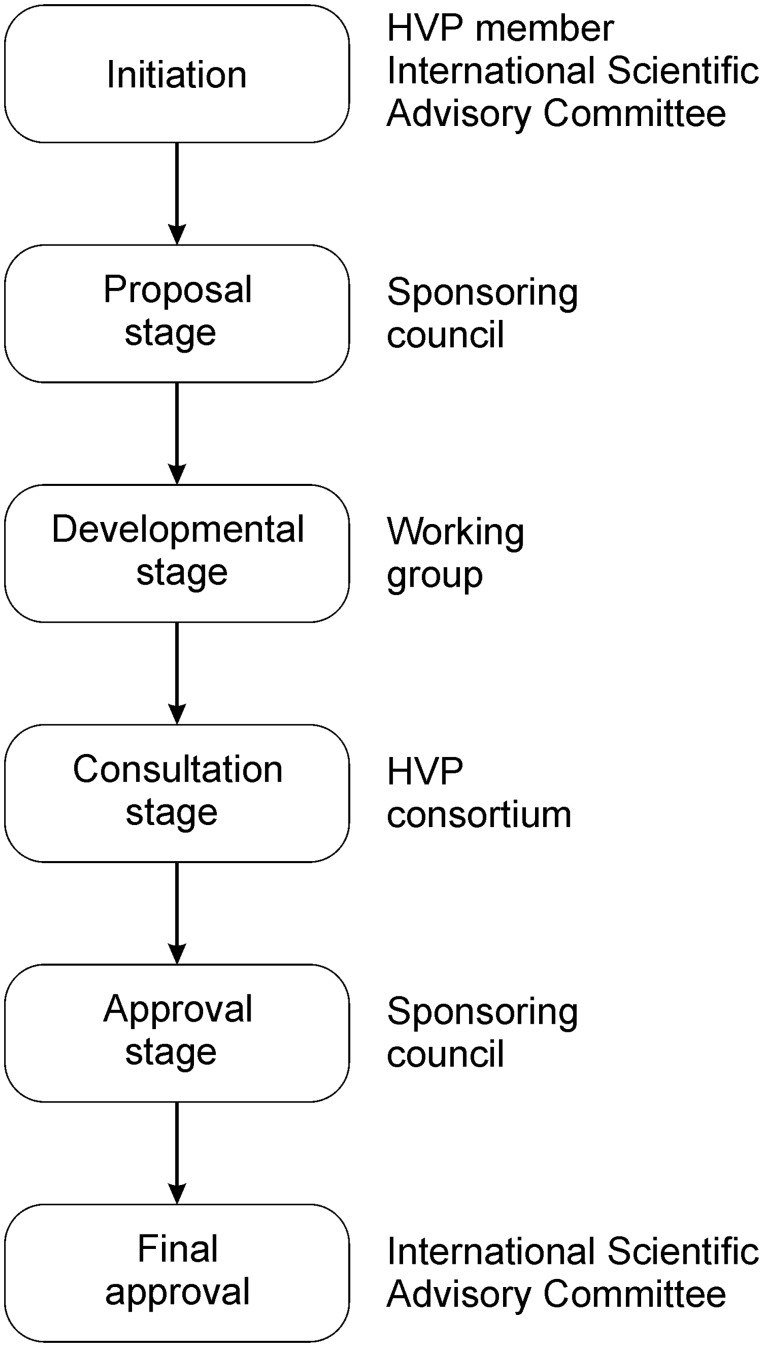
Schema for the steps of the HVP Standards Development Process.

A key component of HVP activities is the development of standards and guidelines. HVP Standards are systems, procedures and technologies that the HVP Consortium has determined must be used by HVP affiliated data sharing infrastructures and should be used by the broader community. HVP guidelines carry less weight and are considered to be beneficial for HVP affiliated data sharing infrastructure and the broader community to adopt. The development process is similar for both of them; see Figure 1 and the HVP Standards Development Process document (http://short.variome.org/PD06-2011).

## Organization of HVP

HVP maintains operational relations with the United Nations Educational, Scientific and Cultural Organization (UNESCO) and the World Health Organization (WHO). HVP members are individuals, entities, governments or organizations interested in the effective sharing of information on genetic variations. Consortium Members possess expertise in clinical genetics, genetic counselling, genetic data collection and/or curation, information technology and administration of scientific projects. The operations of HVP are overseen by a Board of Directors, which currently has six members. The International Scientific Advisory Committee (ISAC; http://short.variome.org/isac) leads the Project in matters of strategic scientific direction for current and future activities. The ISAC is also responsible for overseeing the development and publication of all HVP Standards and Guidelines, as well as the arbitration of the dispute resolution process. The voting members of the ISAC are elected by the two Advisory Councils (six members from each Council) every 2 years, with half the positions on the Committee becoming vacant every 2 years. The Committee can co-opt as many non-voting individuals onto the Committee as it sees fit. The day-to-day work of the Project is facilitated by the staff of the International Coordinating Office (ICO).

The HVP has two advisory councils. The International Confederation of Countries Advisory Council (ICCAC) is the representative body for HVP Country Nodes, which are national organizations that have been established to manage the collection and sharing of variation data directly from diagnostic laboratories within a single country or region. HVP Country Nodes are built, funded and managed by the countries they service.

The Gene/Disease-Specific Database Advisory Council (G/DSDBAC) is the representative body for Gene/Disease-Specific Databases—also called locus-specific variation databases (LSDBs). Gene/disease-specific databases are curated, online collections of information on genetic variations in a single gene, gene family or set of genes implicated in a single disease.

## HVP standards development process

### Initiation

HVP Standards and Guidelines are technical documents that outline best practice techniques, processes and technologies that must or should be used by database managers and curators when storing, manipulating and sharing genetic variation data. They are developed through an open, needs-driven and community-minded process. Requests to initiate the SDP are made to the ISAC by submitting an Activity Proposal (AP), a brief document that outlines:

1. the need the proposed standard or guideline is attempting to address
2. the scope of the proposed activity
3. a plan of action for addressing the activity including suggested Working Group members
4. any resources required
5. the expected deliverables of the project and
6. a recommendation as to whether the final document should be considered for publication as an HVP Standard or HVP Guideline.

Any HVP Consortium member can prepare and submit an AP.

Upon receipt of an AP, the ISAC makes a determination on which of the HVP’s two Advisory Councils should act as Sponsoring Council and manage the SDP for the proposed activity. Activities that deal primarily with country-based data collection and sharing are handled by the ICCAC (http://short.variome.org/iccac), while those that primarily deal with collection and sharing of data on a gene- or disease-specific basis are handled by the G/DSDBAC (http://short.variome.org/gdsdbac). In cases where the proposed Standard or Guideline pertains to both HVP Country Nodes and gene/disease-specific databases, the ISAC may elect to act as the Sponsoring Council.

### Proposal stage

After reviewing the proposed activity, the Sponsoring Council either accepts or rejects the proposal, or refers the AP back to the submitter for clarifications and refinement. A simple majority vote is needed for decision. In case the AP is accepted the Sponsoring Council charters a Working Group to undertake the activity. Working Group members can be any individual who has the necessary expertise or experience to make a substantial contribution.

### Development stage

The Working Group has a maximum of 2 years to develop an exposure draft of the standard or guideline. Working Groups are free to set their own schedule and means of working. The ICO is available to offer support to Working Groups. Their progress is closely monitored by the Sponsoring Council and the Working Group must submit a written report to the Sponsoring Council at every meeting of the Sponsoring Council. The Development stage ends when the Working Group decide to submit their Working Draft for public consultation and the final Working Draft is submitted to the ICO to be edited for style, grammar, concision and clarity.

### Consultation stage

Once the edited draft has been accepted by the Working Group Chair it is released for public comment to the HVP Consortium as an Exposure Draft via the HVP website. The consultation period last for 60 days. All comments are referred back to the Working Group which must address them in a final draft. This Draft for Approval is then submitted to the Sponsoring Council for consideration, accompanied by a detailed accounting by the Working Group Chair of each of the comments received during the Consultation stage and how they have been addressed in the Draft for Approval.

### Approval stage

The Draft for Approval is considered by the Sponsoring Council at their next scheduled meeting. A supermajority of 75% of all members of the Sponsoring Council voting on the matter is required to approve the draft and recommend publication to the ISAC. Failing this, the draft can be referred back to the Working Group for additional work.

### Final approval

Final approval must be obtained from the ISAC. A simple majority is required after which the standard will be published on the HVP website and advocated to the community.

All HVP Standards and Guidelines are published with the Working Group as the designated author and copyright assigned to HVP. After approval the standards and guidelines can be accessed under a Creative Commons Attribution-ShareAlike 4.0 International license (https://creativecommons.org/licenses/by-sa/4.0/). Working Group members may also wish to write an article for an academic journal to draw wider attention to the presence of the HVP Standard or Guideline.

HVP Standards and Guidelines are expected to be reviewed and updated or superseded as new technologies and processes are developed. Any HVP Standard or Guideline can be reviewed at the request of a Consortium member after 2 years have elapsed from the date of publication.

## Current HVP standards and guidelines

The SDP has been recently established. To date two HVP Guidelines have been published. This first HVP Guideline for Gene/Disease-Specific Variant Database Quality Parameters (http://short.variome.org/GL-001-01) specifies quality parameters for summarizing criteria for high quality gene/disease-specific databases. The Guideline is in the process of being implemented into a quality assessment scheme to evaluate the numerous databases available. The other Guideline for Disclaimer Statements for Gene/Disease-Specific Databases (http://short.variome.org/GL-002-01) will be adopted by the major databases, including those in the Leiden Open Variation Database (LOVD) system.

In addition, a number of HVP Standards and Guidelines are under development, including those for:

1. Assigning pathogenicity to a genetic variant
2. Minimal content for gene variant databases (LSDBs)
3. Sequence variant description
4. Disease and phenotype descriptions in gene/disease-specific databases
5. Minimum content of a country-specific variant database
6. Ethics checklist for gene/disease-specific database curators and submitters

The current list is available at http://www.humanvariomeproject.org/solutions/standards-and-guidelines-under-development.html.

## Recommended systems

In parallel to the SDP, the HVP also maintains a process for assessing systems, processes and tools that implement HVP Standards and Guidelines. Recommended System Status is an accreditation process designed to encourage the adoption of HVP Standards and Guidelines and to provide clear guidance to data providers and consumers as to which systems, procedures and tools are known to be in compliance with these standards. Specific systems, processes and tools that have been assessed by HVP are made available free of charge for non-commercial use are eligible for Recommended System Status. Recommended System Status is granted by the HVP ISAC after consideration of the comments and recommendations of at least three peer reviewers.

Applications for Recommended System Status can be made at any time to the ISAC via the ICO. Applications should consist of a concise summary of:

1. the system and its features
2. the extent to which the system is currently being utilized
3. the HVP Standards and Guidelines the system is compliant with and
4. the access and license terms and conditions under which the system is made available.

The applications are reviewed by experts in the field, who are asked to consider:

1. the compatibility of the system with other HVP Recommended Systems
2. the usability and relevance of the system
3. the reliability and robustness of the system
4. the availability of system documentation and training materials and
5. the extent to which the system is currently being utilized.

Recommended systems, processes and tools are listed publicly on the HVP website at http://www.humanvariome project.org/solutions/recommended-systems.html. Recommended systems may publicly display their status by using the phrase ‘a recommended system of the Human Variome Project’ alongside the HVP logo.

Currently, the approved Recommended Systems include Mutalyzer for generating systematic HGVS names for variations (1), Locus Reference Genomic (LRG) sequence format for reference sequences (2), LOVD data management system for LSDBs (3), and HGVS Nomenclature (4). In addition, under review are VarioML—variant data exchange format (5), Variation Ontology (VariO) for systematic description for variation effects, consequences and mechanisms (6) and Cafe Variome, a platform for searching whether data on variants exist rather than sharing data (http://www.cafevariome.org/).

In conclusion, HVP grants three kinds of recommendations. HVP Standards are considered as a must for the community, HVP Guidelines are beneficial for the community to adopt and HVP-Recommended Systems are accredited systems that comply to HVP Standards and Guidelines. A detailed process has been developed for the development of these systems, including community consultation before final approval and publication. Some Standards, Guidelines and Recommended systems have been approved, and several others are under consideration.

*Conflict of interest*. None declared.
